# Enhancing medical care for aniridia through a public health lens: a scoping review of organizational models, patient management, and quality of life assessment

**DOI:** 10.3389/fpubh.2026.1890217

**Published:** 2026-08-21

**Authors:** Marfuga Oteuliyeva, Dastan Kyrykbayev, Gulnar Zhurgumbayeva, Dariga Smailova, Islam Ordabayev, Luca Brusati

**Affiliations:** 1 Kazakh Scientific Research Institute of Eye Diseases, Almaty, Kazakhstan; 2 Kazakh National Medical University named after S.D. Asfendiyarov, Almaty, Kazakhstan; 3 Università degli Studi di Udine Biblioteca Scientifica e Tecnologica, Udine, Italy

**Keywords:** aniridia, health equity, interdisciplinary care, patient-centered care, public health, quality of life, rare diseases, social determinants of health

## Abstract

**Background:**

Congenital aniridia is a rare disorder characterized by partial or complete absence of the iris and is usually associated with other ocular and systemic complications. Its lifelong and multisystem nature creates challenges that extend beyond clinical management and require coordinated, multidisciplinary, and equitable healthcare delivery.

**Methods:**

A scoping review was conducted using the Arksey and O'Malley framework and Joanna Briggs Institute methodology and reported in accordance with PRISMA-ScR. PubMed, Scopus, and Google Scholar were searched for English-language publications from January 2015 to May 2025 addressing organizational models of care, patient-management strategies, and quality-of-life assessment in congenital aniridia. Included sources were descriptively classified by study design and evidence category. Findings were synthesized using a combined deductive and inductive thematic approach.

**Results:**

Fourteen sources met the inclusion criteria and represented different geographical and healthcare settings. The evidence was predominantly observational, retrospective, descriptive, or based on expert and organizational recommendations. Across the reviewed literature, recurrent themes included multidisciplinary coordination, access to specialized expertise, structured referral pathways, registries, telemedicine, lifelong monitoring, genetic counseling, rehabilitation, and patient support. However, direct comparative and longitudinal evaluations of organizational models were lacking. Quality-of-life studies used heterogeneous assessment instruments, limiting direct comparison, although poorer visual function, advanced ocular complications, ocular pain, and broader psychological or systemic burden were generally associated with less favorable patient-reported outcomes.

**Conclusion:**

The available evidence supports the principles of lifelong, multidisciplinary, coordinated, and patient-centered care for congenital aniridia. Future health-system development may benefit from strengthened centers of expertise, standardized referral pathways, registry infrastructure, digital consultation, and improved access to specialized services. Prospective multicentre and longitudinal studies are needed to determine the comparative effectiveness of different organizational models and to standardize patient-reported outcome assessment.

## 1 Introduction

Congenital aniridia is a rare disease characterized by an absent or partially/complete absent of the iris and is commonly associated with reduced visual acuity, nystagmus, and other ocular abnormalities (1, 2). Most cases are associated with mutations or deletions involving the PAX6 gene, which plays a fundamental role in ocular development (2, 3). The resulting phenotype may involve multiple ocular structures and can include glaucoma, foveal and optic nerve hypoplasia, cataract, aniridia-associated keratopathy, corneal opacification, and severe visual impairment (3, 4). Consequently, affected individuals may require lifelong ophthalmic surveillance, visual rehabilitation, and management of progressive ocular complications (1, 5).

The clinical expression of congenital aniridia is heterogeneous. Iris hypoplasia may range from subtle residual iris tissue detectable only by gonioscopy or anterior-segment optical coherence tomography to more apparent structural abnormalities (6). Visual dysfunction extends beyond reduced visual acuity and may include red-green and yellow-blue color-vision deficiencies and abnormalities in retinal function (5, 7). In syndromic forms, particularly WAGR syndrome, aniridia may coexist with Wilms tumor, genitourinary abnormalities, intellectual disability, obesity, hypertension, thyroid abnormalities, and neurodevelopmental disorders (1, 8). Non-ocular manifestations have also been reported in apparently isolated aniridia, further supporting the need for comprehensive clinical assessment (5).

Because of its lifelong and potentially multisystem nature, congenital aniridia presents challenges that extend beyond ophthalmic treatment alone. Patients may require coordinated input from ophthalmologists, geneticists, pediatricians, rehabilitation specialists, psychologists, social workers, and, in syndromic cases, other medical specialists (1, 5). However, access to coordinated care may be limited by fragmented referral pathways, concentration of expertise in a small number of centers, geographical distance, socioeconomic inequalities, and differences in health literacy (9). These barriers may delay diagnosis, interrupt long-term follow-up, and restrict access to genetic assessment, rehabilitation, psychosocial support, and other specialized services (9).

Rare-disease initiatives provide potential organizational frameworks for addressing these challenges. In Europe, policy initiatives have promoted the development of coordinated rare-disease strategies and networks of specialized expertise (10). The European Platform on Rare Disease Registration supports greater interoperability and standardization of registry data (11), while European Reference Networks connect centers of expertise across national borders. The European Reference Network for Rare Eye Diseases (ERN-EYE) provides an example of a network-based approach in which specialist expertise can be shared through virtual multidisciplinary discussions, teleconsultation, and cross-border collaboration while patients continue to receive care within their national healthcare systems (12).

Applied to congenital aniridia, such an organizational approach may involve clearly defined pathways linking primary and pediatric care, regional ophthalmology services, national centers of expertise, genetic and systemic assessment, low-vision rehabilitation, and international expert consultation when required (12). Effective coordination across these levels may help reduce fragmented follow-up, facilitate continuity between pediatric and adult services, and improve access to specialized expertise for patients living far from specialist centers (12). However, the extent to which these organizational mechanisms have been implemented and evaluated specifically for congenital aniridia remains insufficiently documented. Evidence from rare-disease care also indicates that fragmented referral systems and inadequate coordination between specialties can remain important barriers even within established healthcare systems (9).

Population health outcomes are influenced not only by the availability of clinical interventions but also by health-system financing, organizational capacity, and the broader policy environment. International health-system research indicates that healthcare performance and resilience are influenced by the organization and allocation of financial resources under changing macroeconomic conditions (13). Broader public policies outside the healthcare sector may also contribute to population-level health outcomes and health inequalities (14). Although these findings are not specific to congenital aniridia, they support a systems-based perspective in which care for rare diseases is embedded within adequately financed, coordinated, and accessible healthcare structures.

Previous publications have addressed individual components of congenital aniridia care (3, 5). Clinical reviews have primarily summarized genetic mechanisms, ocular manifestations, diagnostic assessment, surveillance, and therapeutic approaches, whereas other sources have emphasized multidisciplinary management, organizational barriers, specialized services, and rare-disease networks (5, 9, 12). Quality-of-life studies have further demonstrated that the burden of congenital aniridia extends beyond clinical measures of visual function and encompasses broader functional and psychosocial dimensions (7, 15). However, these areas have not been comprehensively synthesized within a unified public health framework encompassing organizational models, referral pathways, long-term patient management, and quality-of-life assessment.

Evidence therefore remains fragmented across clinical reviews, observational studies, registries, organizational resources, policy frameworks, and quality-of-life studies. Consequently, it remains unclear which components of existing care models are supported by empirical evidence, which may be transferable across healthcare systems, and where the principal gaps remain in accessibility, continuity of care, multidisciplinary coordination, and patient-centered management.

Accordingly, this scoping review aimed to map and critically synthesize the available evidence on congenital aniridia across three interconnected domains: (1) organizational models of care and referral pathways, (2) long-term patient-management strategies, and (3) approaches to quality-of-life assessment. The review examined how multidisciplinary and lifelong care is organized, which barriers affect equitable access and continuity of services, and which instruments and outcomes have been used to evaluate quality of life.

## 2 Method

### 2.1 Research methodology

This study used a scoping review approach based on the framework developed by Arksey and O'Malley (16) and subsequent methodological guidance for scoping reviews provided by the Joanna Briggs Institute (JBI) (17). Reporting followed the Preferred Reporting Items for Systematic Reviews and Meta-Analyses extension for Scoping Reviews (PRISMA-ScR) (18).

A scoping review design was selected because the literature on congenital aniridia relevant to healthcare organization, long-term patient management, and quality of life is heterogeneous and includes different study designs, clinical reviews, registry-based studies, guidelines, and organizational or policy-related sources. This approach enabled systematic mapping of the available evidence, identification of recurring concepts and organizational approaches, and characterization of gaps in the existing literature without restricting the review to a single narrowly defined clinical intervention or outcome.

The literature search covered publications from January 2015 to May 2025. This period was selected to capture contemporary developments in the clinical management of congenital aniridia, rare-disease care organization, multidisciplinary care, registry infrastructure, and quality-of-life assessment.

### 2.2 Method of data retrieval and sources

Systematic literature searches were conducted in SCOPUS, PubMed, and Google Scholar to identify publications relevant to the biomedical, clinical, and public health dimensions of congenital aniridia care. The search strategy combined controlled vocabulary terms and free-text keywords related to congenital aniridia and the three predefined domains of the review: healthcare organization and referral pathways, patient-management strategies, and quality of life.

The complete PubMed search strategy was as follows: (“Aniridia” [MeSH Terms] OR aniridia [Title/Abstract] OR “congenital aniridia” [Title/Abstract] OR “PAX6-related aniridia” [Title/Abstract] OR “WAGR syndrome” [Title/Abstract]) AND (“Delivery of Health Care” [MeSH Terms] OR “Patient Care Management” [MeSH Terms] OR “Continuity of Patient Care” [MeSH Terms] OR “Referral and Consultation” [MeSH Terms] OR “Registries” [MeSH Terms] OR “Telemedicine” [MeSH Terms] OR “Quality of Life” [MeSH Terms] OR “Rehabilitation” [MeSH Terms] OR “public health” [Title/Abstract] OR “health system” [Title/Abstract] OR “health systems” [Title/Abstract] OR “healthcare system” [Title/Abstract] OR “healthcare systems” [Title/Abstract] OR “care model” [Title/Abstract] OR “care models” [Title/Abstract] OR “organizational model” [Title/Abstract] OR “organizational models” [Title/Abstract] OR “organizational model” [Title/Abstract] OR “organizational models” [Title/Abstract] OR “integrated care” [Title/Abstract] OR “multidisciplinary care” [Title/Abstract] OR “interdisciplinary care” [Title/Abstract] OR “patient management” [Title/Abstract] OR “care pathway” [Title/Abstract] OR “care pathways” [Title/Abstract] OR “referral pathway” [Title/Abstract] OR “referral pathways” [Title/Abstract] OR “center of expertise” [Title/Abstract] OR “centers of expertise” [Title/Abstract] OR “center of expertise” [Title/Abstract] OR “centers of expertise” [Title/Abstract] OR registry [Title/Abstract] OR registries [Title/Abstract] OR telemedicine [Title/Abstract] OR “quality of life” [Title/Abstract] OR HRQoL [Title/Abstract] OR “patient-reported outcome” [Title/Abstract] OR “patient-reported outcomes” [Title/Abstract] OR rehabilitation [Title/Abstract]) AND (“2015/01/01” [Date - Publication]: “2025/05/31” [Date - Publication]).

The search syntax was adapted to the indexing systems and search functions of Scopus and Google Scholar while retaining the same principal concepts. Boolean operators (AND/OR), database-specific field specifications, and publication-date restrictions were applied as appropriate. The initial search was performed with no restrictions limits to maximize retrieval. However, only English-language sources were eligible for inclusion in the final review. Further documents were obtained by manually checking the reference lists of key studies and relevant reviews.

### 2.3 Eligibility criteria

Sources were eligible if they focused on individuals with congenital aniridia, including isolated PAX6-related aniridia and syndromic forms such as WAGR syndrome, and addressed at least one of the three predefined review domains: (1) organizational models of care and referral pathways, (2) patient-management strategies, or (3) health-related quality of life. Sources were not required to address all three domains. Publications addressing only one or two domains were included when the relevant topic constituted a substantive component of the objectives, methods, results, or recommendations rather than a brief incidental mention. Only information directly relevant to the predefined review domains was extracted from such sources.

Eligible source types included observational studies, registry-based studies, retrospective case series, mixed-methods studies, narrative and systematic reviews, clinical guidelines, consensus statements, and policy or organizational reports containing relevant and extractable information on congenital aniridia care. Mixed-methods studies were eligible when findings related to healthcare organization, patient management, or quality of life could be clearly identified and extracted. Narrative reviews and guidance documents were included when they provided structured recommendations, described care pathways, or synthesized evidence directly relevant to one or more of the predefined review domains.

Sources were excluded if they focused exclusively on molecular mechanisms, basic genetics, laboratory research, or animal models without a direct patient-care or health-system component. Publications were also excluded when congenital aniridia or one of the predefined review domains was mentioned only incidentally without relevant extractable information. Editorials, letters, duplicate publications, non-English-language publications, and conference abstracts lacking sufficient methodological or outcome information for meaningful data extraction were excluded.

### 2.4 Process of selecting relevant studies

A total of 1,235 records were identified: PubMed (*n* = 287), Scopus (*n* = 418), Google Scholar (*n* = 520), and manual reference-list screening (*n* = 10). After combining the retrieved records, 230 duplicates were identified and removed, leaving 1,005 unique records for title and abstract screening.

Two reviewers independently screened the titles and abstracts according to the predefined eligibility criteria. Full texts of 85 potentially eligible publications were subsequently assessed independently by the same reviewers. Disagreements at either stage were resolved through discussion, with consultation of a third reviewer when consensus could not be reached. Following full-text assessment, 14 sources met the eligibility criteria and were included in the final synthesis. Reasons for full-text exclusion are summarized in the study-selection flow diagram.

**Figure 1:**
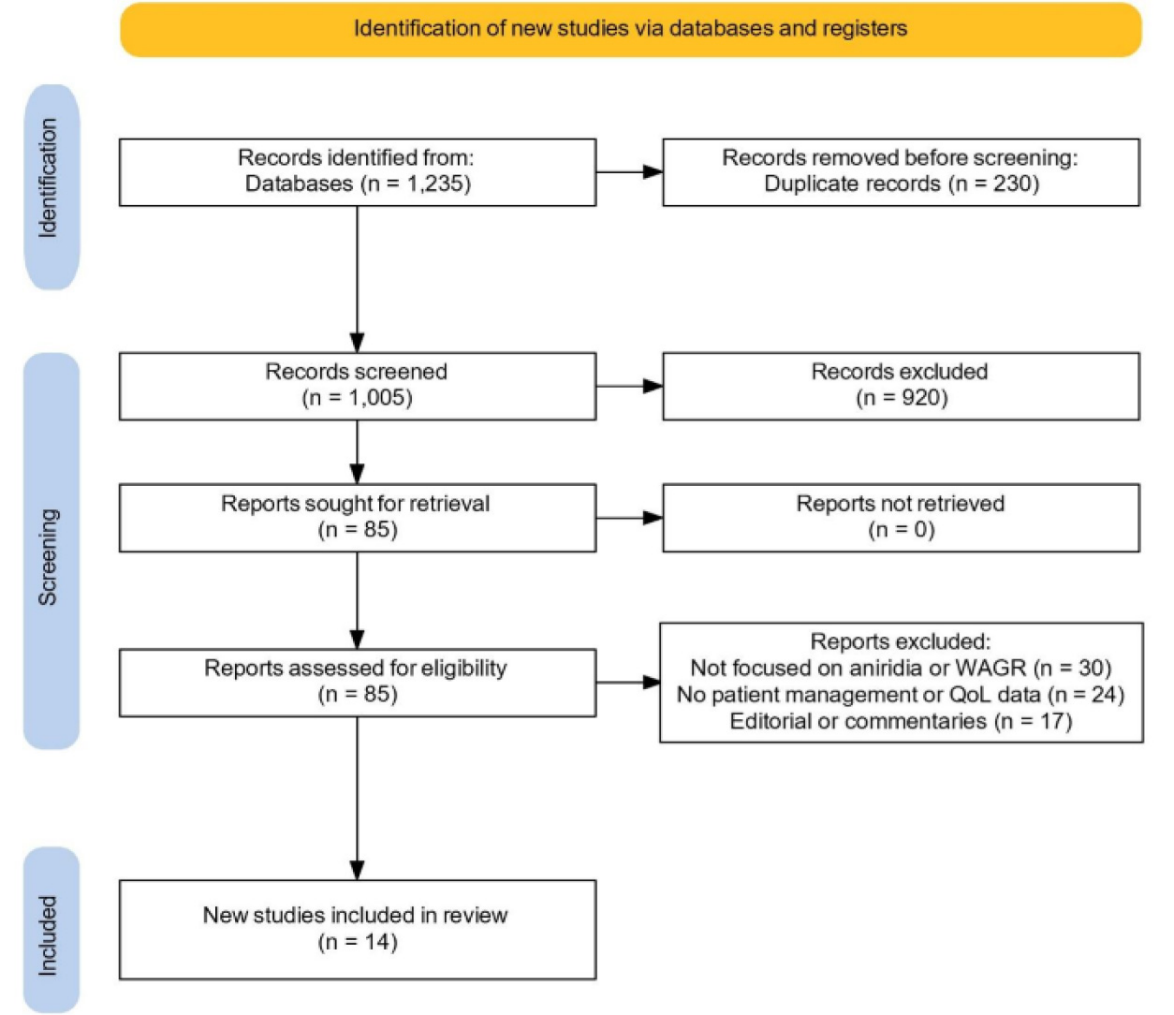
PRISMA-ScR flow diagram illustrating the selection process for studies included in the scoping review.

The study-selection process is presented in the PRISMA-ScR flow diagram in Figure 1 (18).

### 2.5 Data extraction and synthesis

A customized and pilot-tested data-charting form was used to ensure consistent extraction of relevant information. For each included source, the reviewers extracted the authors, year of publication, country or geographical setting, study design, source type, sample characteristics, thematic focus, main findings, and principal methodological limitations. For sources assessing quality of life, additional information was extracted on the measurement instruments, participant characteristics, and reported patient-reported outcomes.

Consistent with the objectives of a scoping review, a formal risk-of-bias assessment was not undertaken. Instead, to facilitate interpretation of the strength and nature of the available evidence, each included source was descriptively classified according to study design and evidence category. Categories included cross-sectional observational studies, registry-based studies, retrospective case series, mixed-methods or expert-survey studies, narrative or clinical reviews, clinical guidelines, policy frameworks, organizational reports, and patient-advocacy resources. Where applicable, sample size, data-collection method, presence of a comparator, and major methodological limitations were also recorded. This classification was used to distinguish primary empirical evidence from secondary evidence, expert recommendations, policy proposals, and descriptive organizational information. No numerical quality score or formal hierarchy of evidence was applied.

The thematic synthesis used a combined deductive and inductive approach. Three overarching domains–organizational models of care and referral pathways, patient-management strategies, and quality-of-life assessment–were defined deductively *a priori* based on the review objectives and research questions. During data extraction, findings from each source were coded within one or more of these predefined domains. Within each domain, recurrent concepts and subthemes were subsequently identified inductively, including centralized care, multidisciplinary coordination, referral pathways, registries, telemedicine, long-term clinical monitoring, genetic counseling, rehabilitation, and patient-reported outcomes.

Sources addressing more than one domain were coded within all relevant categories. The reviewers compared the coded findings, discussed discrepancies, and refined the thematic structure by consensus. The final narrative synthesis examined areas of convergence and divergence across the included sources and identified evidence gaps in relation to study design, healthcare setting, organizational models, patient-management strategies, and quality-of-life assessment.

### 2.6 Ethical considerations

Ethical approval was not required because this scoping review used exclusively previously published and publicly available data and involved no recruitment of participants, collection of new individual-level data, or direct interaction with patients. No identifiable personal information was collected or analyzed.

## 3 Results

### 3.1 Characteristics of included studies

Fourteen sources published between 2015 and 2025 met the eligibility criteria and represented diverse geographical and healthcare settings. The included literature comprised primary empirical studies as well as narrative and clinical reviews, guideline and policy sources, and organizational resources. Sources were mapped to one or more of the three predefined review domains: organizational models of care and referral pathways, patient-management strategies, and quality-of-life assessment.

Of the 14 included sources, nine provided primary empirical data. These comprised six cross-sectional questionnaire, survey, or observational studies, one retrospective registry-based study, and two retrospective interventional case series. The remaining five sources consisted of two narrative or clinical reviews, one policy framework, one expert and patient-perspective guideline chapter, and one patient-advocacy organizational resource. Only one observational study included a comparator group, and no randomized controlled trials, prospective comparative studies, or longitudinal evaluations of organizational care models were identified. Consequently, the available evidence was predominantly descriptive, cross-sectional, retrospective, or based on expert and organizational recommendations. Although these sources were useful for mapping existing care practices, organizational approaches, and policy perspectives, their designs provided limited evidence for causal relationships or for the comparative effectiveness of specific models of congenital aniridia care.

The main characteristics of the included sources, including study design, thematic focus, evidence category, key findings, and principal methodological limitations, are summarized in Table 1.

**Table 1:** Characteristics, evidence categories, and methodological limitations of the included sources.

Author	Year	Country	Study design	Focus	Sample	Evidence type	Key findings	Subtheme	Main limitation
Tripathy and Salini (1)	2023	Global	Clinical overview	Clinical algorithms	N/A	Narrative clinical reference/secondary evidence	Standardized screening schedule for glaucoma & WAGR.	Patient management	No original sample; not a systematic review and no formal risk-of-bias appraisal.
Landsend et al. (5)	2021	Norway	Narrative review	Integrated models	N/A	Narrative review/secondary evidence	Rare-disease centers improve surveillance & rehab timelines.	Organizational model	No original sample; narrative review without a reported systematic search or formal quality appraisal.
Pedersen et al. (7)	2018	Norway	Quantitative	Color vision	36 patients with aniridia + 52 controls; 31 patients in the primary analysis	Cross-sectional controlled observational study	Color vision loss in patients with aniridia.	QoL	Small, single-country, cross-sectional cohort; 5 patients were excluded from the primary analysis because of congenital color-vision genotypes.
Duffy et al. (8)	2021	USA	Registry	WAGR spectrum	91 registry participants	Cross-sectional rare-disease registry questionnaire study	High burden of fatigue & caregiver stress.	Patient management	Self-reported registry data were not verified against medical records; selection bias was possible, and ages at survey completion were unavailable.
Gunes et al. (9)	2025	Türkiye	Mixed method	System barriers	21 specialists from 16 pediatric subspecialties	Cross-sectional online expert survey	Fragmented referral pathways; policy solutions proposed.	Organizational model	Small sample from a single university hospital; findings may not be generalisable beyond that setting.
Hoxha et al. (15)	2025	Germany	Cross-sectional observational study	QoL assessment	71 participants (27 children, 44 adults)	Retrospective cross-sectional survey-based study	Quality of life was lower in patients with advanced keratopathy or early cataract surgery.	QoL	Low response rate (34.3%); self-reported data, no normal-vision control group, and clinical data did not always coincide with questionnaire timing.
ERN- EYE (12).	2024	EU	Policy framework	Cross-border care	N/A	European policy and organizational framework	Virtual case boards & telemedicine to share expertise.	Organizational model	Non-empirical source; describes network structures but does not comparatively evaluate patient or health-system outcomes.
Inokuchi et al. (19)	2020	Japan	Cross-sectional observational study	QoL assessment with NEI-VFQ-25	37 patients (73 eyes)	Cross-sectional questionnaire study (conference abstract)	Lower visual acuity in the dominant eye was associated with reduced quality of life in aniridia patients.	QoL	Conference abstract only; limited detail on sampling, methods, and study limitations.
Aniridia Foundation Int. (24)	2025	Global	Web resource	Advocacy	N/A	Patient-advocacy/organizational web resource	Provides navigation & peer-mentor programmes.	Organizational model	Non-peer-reviewed, non-empirical web resource; no formal methods or outcome evaluation.
Landsend et al. (20)	2024	Norway	Cross-sectional observational study	HRQoL	29 adults with congenital aniridia	Cross-sectional HRQoL questionnaire study	Adults with congenital aniridia scored worse on certain measures of HRQoL than the general population	QoL	Small adult-only Norwegian sample and cross-sectional design, limiting generalisability and causal inference.
Obst et al. (21)	2025	Germany	Cross-sectional analysis of registry data	Multi-system phenotyping; comorbidity profiling.	337 patients	Retrospective single-center registry study	High rate of comorbidities. Reveals the need for multidisciplinary care and integrated referral pathways.	Organization al model	Retrospective, monocentric design; genetic testing was available for only 187/337 patients, and exact age at initial diagnosis was unavailable.
Poli et al. (25)	2015	Europe	Guideline report	Development of multidisciplinary guidelines and patient advocacy role	N/A (book chapter/guideline perspective)	Expert and patient-perspective guideline chapter	Recommends center-based multidisciplinary teams for lifelong care	Organizational models.	Non-empirical book chapter; recommendations were not supported by a reported systematic evidence appraisal or outcome evaluation.
Qui et al. (22)	2016	Europe	Retrospective case series	Evaluate outcomes of BDI lens implantation for congenital aniridia	15 patients (23 eyes)	Retrospective interventional case series	70% achieved BCVA > 20/200 with reduced photophobia	Patient management	Small retrospective case series without a control group; only 15 patients (23 eyes) were included.
Rickmann et al. (23)	2016	Europe	Retrospective interventional case series	Prosthetic iris implantation outcomes	34 patients	Retrospective interventional case series	Improved glare/cosmesis and visual function	Patient management	Retrospective case series with a small sample and no control group.

The findings of this review should be interpreted in light of several important limitations of the available evidence. The evidence base was dominated by small cross-sectional studies, retrospective registry analyses, uncontrolled case series, narrative reviews, and non-empirical policy or organizational resources. Multicentre evidence was very limited, and no prospective multicentre comparative studies evaluating different models of congenital aniridia care were identified. In addition, substantial methodological heterogeneity was observed across studies in terms of study design, sample size, participant characteristics, healthcare setting, outcome measures, and methods of quality-of-life assessment (7–9, 15, 19–23).

Importantly, no randomized controlled trials, prospective comparative studies, or longitudinal evaluations of organizational care models were identified. Consequently, the available literature allows recurrent organizational principles, patient-management needs, and quality-of-life concerns to be mapped but does not permit robust conclusions regarding causal relationships, long-term effectiveness, or the comparative superiority of centralized, regional, network-based, or hybrid models of care. Recommendations concerning centers of expertise, multidisciplinary coordination, telemedicine, registries, structured referral pathways, and patient navigation should therefore be regarded as evidence-informed and hypothesis-generating rather than as definitively validated interventions.

### 3.2 Organizational models for aniridia care

The included sources described several organizational approaches relevant to congenital aniridia care, including centralized rare-disease centers, center-based multidisciplinary services, cross-border expert networks, registry-supported care, and patient-led navigation and advocacy models (5, 9, 12, 21, 24, 25). However, none of the included sources directly compared these organizational approaches using standardized clinical, patient-reported, accessibility, economic, or health-system outcomes. Therefore, the identified models were compared descriptively according to their organizational structure, principal functions, potential advantages, implementation limitations, and potentially transferable components.

A comparative overview of the main organizational models identified in the literature, including their principal characteristics, potential advantages, limitations, and transferable elements, is presented in Table 2.

**Table 2:** Comparative synthesis of organizational models relevant to aniridia care.

Model and setting	Main organizational components	Potential advantages	Main limitations of the model or evidence	Potentially transferable elements
Rare-disease center model, Norway—Landsend et al. (5)	Centralized expertise; multidisciplinary surveillance; rehabilitation; coordinated long-term follow-up	Continuity of care; concentration of expertise; integration of clinical and rehabilitative services	Based mainly on narrative secondary evidence; no direct comparative outcome evaluation; requires specialist resources	Designated centers, standardized follow-up, integrated rehabilitation
Center-based multidisciplinary model, Türkiye—Gunes et al. (9)	Coordination between primary, secondary, and tertiary care; multidisciplinary team; structured referrals	May reduce fragmentation and clarify professional responsibilities	Small expert survey from one university hospital; not exclusively focused on aniridia; proposed rather than evaluated model	Defined referral criteria, care coordinator, multidisciplinary case review
Cross-border network model, European Union—ERN-EYE (12)	Centers of expertise; virtual case boards; telemedicine; cross-border consultation	Expands access to rare-disease expertise; supports complex cases; may reduce travel	Non-empirical organizational source; dependent on digital infrastructure and network participation; no comparative aniridia-specific outcomes	Virtual multidisciplinary boards, teleconsultation, shared expertise
Patient-led navigation and advocacy model, international—Aniridia Foundation International (24)	Patient education; peer support; advocacy; navigation; psychosocial support	Addresses information, engagement, and psychosocial barriers	Non-peer-reviewed organizational resource; no formal outcome evaluation	Patient navigation, peer mentorship, co-design with patient organizations
Registry-supported model, Germany—Obst et al. (21)	Registry-based phenotyping; identification of systemic comorbidities; support for multidisciplinary referral	Generates structured data for surveillance, research, and service planning	Retrospective single-center design; incomplete genetic testing; care outcomes not evaluated	Disease registry, standardized datasets, registry-triggered referrals
Multidisciplinary guideline model, Europe—Poli et al. (25)	Lifelong specialist care; multidisciplinary teams; patient-organization involvement	Provides structured recommendations and incorporates patient perspectives	Non-empirical guidance; no reported systematic evidence appraisal or outcome comparison	Lifelong pathway, patient participation, multidisciplinary standards

Norwegian model described by Landsend et al. (5) emphasized the concentration of expertise within rare-disease centers, where ophthalmic surveillance, rehabilitation, and multidisciplinary follow-up may be coordinated within a unified care pathway. Its principal potential advantages include continuity of care, concentration of specialist expertise, and integration of clinical and rehabilitation services. Nevertheless, the supporting publication was a narrative review rather than a comparative evaluation and did not provide quantitative evidence that centralized care resulted in better outcomes than decentralized care.

In Türkiye, Gunes et al. described fragmentation between primary, secondary, and tertiary levels of care and proposed a center-based multidisciplinary approach to strengthen referral pathways and professional coordination (9). However, this model was derived from an expert survey conducted within a single institutional setting and was not prospectively evaluated using patient or health-system outcomes.

ERN-EYE represents a network-based approach in which national centers remain responsible for direct patient care, while virtual multidisciplinary case discussions, telemedicine, and cross-border consultation provide access to specialized expertise (12). Unlike a fully centralized national model, this structure may be particularly relevant in settings where the small number and geographical dispersion of patients make it impractical to establish a comprehensive aniridia center in every region. Potential limitations include dependence on digital infrastructure, institutional participation, interoperability, and clearly defined referral procedures.

The Aniridia Foundation International provides a complementary patient-led model based on advocacy, education, peer support, and patient navigation (24). Unlike specialist-center and cross-border network models, its primary function is not the delivery of direct clinical care but the reduction of informational, psychosocial, and navigation barriers and the promotion of patient and family involvement. However, the available information is derived from an organizational web resource, and formal evaluations of its effects on healthcare access, adherence, quality of life, or clinical outcomes were not identified.

Registry-based evidence from Germany provides another complementary organizational component. Obst et al. demonstrated a substantial burden of ocular and systemic comorbidities among patients included in the Homburg Registry for Congenital Aniridia, supporting the rationale for multidisciplinary surveillance and integrated referral pathways (21). Nevertheless, the study was retrospective and single-center and did not establish whether participation in the registry itself improved healthcare delivery or patient outcomes.

The European guideline framework presented by Poli et al. similarly supported lifelong multidisciplinary care and the involvement of patients and patient organizations in care planning (25). However, these recommendations were based primarily on expert and patient perspectives and were not supported by a reported systematic comparison of alternative organizational models or by formal outcome evaluation.

Despite differences between these approaches, several recurring organizational components were identified: designated specialist expertise, multidisciplinary coordination, structured referral pathways, lifelong surveillance, genetic and systemic assessment, rehabilitation, registry support, digital consultation, and meaningful patient involvement (5, 9, 12, 21, 24, 25). These components may represent potentially transferable elements of organized congenital aniridia care. However, their comparative effectiveness remains uncertain because no prospective studies directly comparing centralized, regional, network-based, or hybrid organizational models were identified.

### 3.3 Integrative synthesis of patient-management strategies for aniridia care

Across the included sources, there was broad agreement that congenital aniridia requires lifelong, multidisciplinary, and risk-adapted management rather than isolated ophthalmic interventions. The proposed components of care generally included regular ophthalmic surveillance, assessment of systemic and syndromic manifestations, genetic evaluation and counseling, visual rehabilitation, psychosocial support, and coordination between pediatric, adult, and specialist services (1, 5, 8, 9, 21, 25).

With regard to clinical monitoring, the included sources consistently emphasized surveillance for glaucoma, cataract, aniridia-associated keratopathy, progressive visual impairment, and systemic complications associated with syndromic forms such as WAGR syndrome (1, 5, 8, 21, 25). Some clinical sources proposed structured screening schedules, particularly for glaucoma and Wilms tumor risk (1, 8). However, the included literature did not provide a uniform evidence-based consensus regarding the exact frequency of ophthalmic, genetic, renal, or other systemic evaluations across all age groups. The available evidence therefore supports structured and age- or risk-adapted follow-up but not a single universally validated monitoring schedule.

Genetic assessment and counseling were repeatedly identified as important components of patient management because they may help distinguish isolated PAX6-related aniridia from syndromic forms, guide systemic surveillance, support family counseling, and inform assessment of relatives (1, 5, 8, 25). Nevertheless, no included study directly compared different models of genetic service delivery or evaluated whether systematic genetic counseling improved long-term clinical or patient-reported outcomes.

Transition from pediatric to adult care was recognized as an important component of lifelong management, particularly because ocular complications and systemic comorbidities may evolve over time (5, 12, 21, 25). However, the included sources provided limited operational detail regarding the optimal age of transfer, responsibility for coordinating transition, use of standardized transition plans, or outcomes of structured transition programmes. This represents an important gap in the current evidence.

Psychosocial and family-centered care constituted another recurring component of management. Registry findings demonstrated substantial fatigue and caregiver burden among families affected by WAGR spectrum disorders (8), while patient-advocacy resources emphasized education, peer support, patient navigation, and active participation of patients and families in long-term care (24). These approaches complement clinical monitoring by addressing informational, emotional, and social barriers. However, their effects on treatment adherence, healthcare access, quality of life, and continuity of care have not been formally evaluated.

Surgical interventions addressed specific visual and functional consequences of iris deficiency. Qiu et al. reported improved visual function and reduced photophobia following black diaphragm intraocular lens implantation (22), whereas Rickmann et al. described improvements in glare, cosmesis, and visual function after prosthetic iris implantation (23). However, both studies were retrospective uncontrolled case series with relatively small samples. Their findings support the potential role of surgical intervention in selected patients but should not be interpreted as evidence for a comprehensive long-term management strategy.

Taken together, the included evidence supports a multidimensional care pathway combining ophthalmic surveillance, systemic risk assessment, genetic counseling, rehabilitation, psychosocial support, patient and family involvement, and coordinated transition between pediatric and adult services (1, 5, 8, 9, 12, 21, 24, 25). Nevertheless, consensus was substantially stronger regarding the essential components of care than regarding their optimal timing, frequency, organization, or comparative effectiveness. Prospective studies are needed to evaluate standardized monitoring schedules, structured transition programmes, multidisciplinary care pathways, and patient- and family-centered interventions using clinical, patient-reported, accessibility, and health-system outcomes.

### 3.4 Comparative synthesis of quality-of-life assessment

Quality of life and related functional outcomes in individuals with congenital aniridia were assessed in four included studies using substantially different measurement approaches (7, 15, 19, 20). The instruments used, domains assessed, study populations, principal findings, and implications for comparability are summarized in Table 3.

**Table 3:** Quality of life assessment.

Author	Year	Instrument	Type of assessment	Sample	Main domain assessed	Key findings	Implications for comparability
Pedersen et al. (7)	2018	CAD test	Objective visual-function assessment	36 participants	Color vision	Quantifiable color-vision impairment in aniridia	Measures a specific visual function rather than QoL directly; not directly comparable with questionnaire-based HRQoL scores
Hoxha et al. (15)	2025	ANIRIDIA-NET survey	Aniridia-focused survey	71 participants	Disease-related QoL and functional impact	Lower QoL in patients with advanced keratopathy or early cataract surgery; many remained independent with visual aids	Disease-focused assessment differs from both generic and vision-specific instruments
Inokuchi et al. (19)	2020	NEI VFQ-25 survey	Vision-specific patient-reported questionnaire	37 patients	Vision-related functioning	Worse visual acuity in the dominant eye was associated with eye pain, poorer distance vision, and reduced social function	Captures vision-related QoL but cannot be directly compared with generic HRQoL instruments
Landsend et al. (20)	2024	SF-36 & EQ visual analog scale (VAS)	Generic HRQoL assessment	29 adults	General physical and psychological health	Poorer HRQoL associated with anxiety, depression, obesity and ocular pain	Captures broader health burden but may reflect dimensions not assessed by vision-specific tools

The included studies used substantially different approaches to assess quality of life and related functional outcomes. Pedersen et al. used the Color Assessment and Diagnosis (CAD) test, which measures color-vision impairment rather than quality of life directly (7). Inokuchi et al. used the NEI VFQ-25 to assess vision-related functioning (19), Hoxha et al. applied the ANIRIDIA-NET survey to evaluate disease-related quality of life (15), and Landsend et al. used the SF-36 and EQ visual analog scale to assess broader physical and psychological aspects of health-related quality of life (20).

This methodological heterogeneity limits direct comparison across studies because the instruments measure different constructs. Nevertheless, poorer visual function, advanced ocular complications, ocular pain, and greater psychological or systemic burden were generally associated with less favorable quality-of-life outcomes (7, 15, 19, 20). Differences in sample size, patient characteristics, disease severity, and measurement instruments precluded meaningful pooling of the reported outcomes.

Overall, the available evidence indicates that congenital aniridia affects multiple dimensions of functioning and wellbeing beyond visual acuity alone. Future studies would benefit from standardized outcome measures combining vision-specific patient-reported outcomes with broader health-related quality-of-life assessment and, where appropriate, an aniridia-specific instrument.

### 3.5 Public health and health-system implications

From a public health perspective, improving congenital aniridia care requires organizational approaches extending beyond individual clinical interventions. Recurring health-system priorities identified across the included sources included designated centers of expertise, rare-disease registries, standardized referral and long-term follow-up pathways, and stronger coordination across primary, secondary, and tertiary care (5, 9, 12, 21, 25).

Centers of expertise may facilitate concentration of specialist knowledge, multidisciplinary assessment, genetic and systemic evaluation, rehabilitation, and continuity of lifelong follow-up (5, 21, 25). Where establishing multiple highly specialized centers is not feasible, network-based approaches such as ERN-EYE illustrate how teleconsultation, virtual multidisciplinary case discussions, and cross-border exchange of expertise may complement national referral systems (12).

Rare-disease registries may support longitudinal surveillance, characterization of disease burden and systemic comorbidities, research, service planning, and development of evidence-informed management pathways (8, 21, 26–30). Their value, however, depends on standardized data collection, appropriate governance, interoperability, and integration with routine clinical care.

The reviewed evidence also supports the development of structured clinical and referral pathways that define responsibilities across different levels of care (1, 5, 9, 25). Such pathways may integrate ophthalmic surveillance, genetic counseling, systemic assessment, rehabilitation, psychosocial support, and transition from pediatric to adult services. Digital consultation and patient-navigation mechanisms may further improve access to specialized expertise, particularly for patients living far from specialist centers (12, 24).

Overall, congenital aniridia should be considered not only a rare ophthalmic disorder but also a health-system coordination challenge. A potentially transferable framework would combine designated specialist expertise, structured referral pathways, multidisciplinary lifelong care, registry infrastructure, digital consultation, and meaningful involvement of patients and patient organizations (5, 8, 9, 12, 21, 24, 25). These elements should be regarded as evidence-informed priorities for future implementation and evaluation rather than as organizational interventions of established comparative superiority.

### 3.6 Cross-study critical synthesis

Across the included literature, substantial heterogeneity was observed in study design, population, healthcare setting, and outcome assessment. Nevertheless, several areas of convergence emerged. Sources addressing organizational and clinical care consistently emphasized lifelong multidisciplinary management, coordinated referral pathways, and access to specialized expertise (5, 9, 12, 21, 25). Registry-based and organizational sources additionally highlighted systemic assessment, rehabilitation, psychosocial support, patient navigation, and meaningful patient involvement (8, 21, 24).

Differences between organizational models largely reflected healthcare context rather than conflicting principles of care. The Norwegian literature emphasized centralized rare-disease expertise and coordinated rehabilitation (5), the Turkish evidence focused on fragmentation between levels of care and referral coordination (9), ERN-EYE illustrated a network-based model using virtual consultation and cross-border expertise (12), and patient organizations emphasized education, navigation, peer support, and patient participation (24). However, none of these approaches has been directly compared using standardized clinical or health-system outcomes.

Similar variation was evident in patient-management and quality-of-life evidence. Clinical and review-based sources emphasized surveillance for ocular and systemic complications, genetic assessment, and multidisciplinary follow-up (1, 5, 21, 25), whereas registry and advocacy sources placed greater emphasis on caregiver burden, psychosocial needs, and patient support (8, 24). Quality-of-life studies used heterogeneous instruments assessing different dimensions of visual function and health-related wellbeing (7, 15, 19, 20), limiting direct comparison across studies.

Overall, the evidence demonstrates stronger agreement regarding the essential components of care than regarding their optimal organization, timing, or comparative effectiveness. Small sample sizes, predominantly cross-sectional or retrospective designs, methodological heterogeneity, and limited multicentre and longitudinal evidence restrict causal inference and comparison between organizational models. The findings therefore support a common set of care principles but do not establish the superiority of any single centralized, regional, network-based, or hybrid model.

## 4 Discussion

The findings of this review indicate that congenital aniridia should be considered not only as a lifelong ophthalmic condition but also as a health-system coordination challenge. Although clinical management remains central, effective long-term care may additionally require genetic assessment, systemic surveillance, rehabilitation, psychosocial support, structured transition between pediatric and adult services, and active participation of patients and families (5, 8, 21, 24, 25).

The organization of these services should be adapted to national and regional healthcare structures. Designated centers of expertise may facilitate multidisciplinary coordination and continuity of care, whereas network-based models supported by telemedicine and virtual specialist consultation may provide a feasible alternative where patients and expertise are geographically dispersed (12). Rare-disease registries may further support surveillance, research, service planning, and standardized data collection (21, 26–30). However, these approaches should be regarded as evidence-informed organizational priorities rather than as models of proven comparative superiority.

Equitable access should remain an important consideration. Geographical distance, socioeconomic circumstances, and differences in access to healthcare services may affect continuity of specialist care and rehabilitation (31–33). Rare diseases may also impose substantial direct and indirect financial burdens on patients, families, and healthcare systems (34, 35). These findings reinforce the importance of incorporating accessibility, rehabilitation, caregiver needs, and patient participation into the planning of long-term congenital aniridia care.

Several limitations should be acknowledged. The included evidence was heterogeneous in study design, population, healthcare setting, and outcome assessment, and most studies were small, cross-sectional, retrospective, or descriptive. Only English-language sources were included, and gray literature was not systematically searched across all possible repositories. In addition, the absence of prospective comparative and longitudinal studies limits conclusions regarding the effectiveness of specific organizational models.

## 5 Conclusion

This scoping review indicates that the available literature consistently supports the principles of lifelong, multidisciplinary, and coordinated care for congenital aniridia. Recurrent components include access to specialized expertise, structured referral pathways, ophthalmic and systemic surveillance, genetic assessment and counseling, rehabilitation, psychosocial support, and meaningful involvement of patients and families (5, 8, 9, 12, 21, 24, 25). However, the supporting evidence is predominantly observational, retrospective, descriptive, or based on expert and organizational recommendations, and direct comparative or longitudinal evaluations of alternative care models remain lacking.

Based on the available evidence, future health-system development may benefit from designated centers of expertise, standardized clinical and referral pathways, stronger coordination across levels of care, rare-disease registries, and national and international professional networks (5, 9, 12, 21, 25). Registry infrastructure may support longitudinal surveillance, standardized data collection, service planning, and research collaboration (21, 26–30). Digital technologies, including telemedicine and virtual multidisciplinary consultation, may further improve access to specialist expertise, particularly for geographically remote patients (12).

Future research should prioritize prospective multicentre and longitudinal studies comparing centralized, regional, network-based, and hybrid models of care using standardized clinical, patient-reported, accessibility, economic, and health-system outcomes. Further studies are also needed to define optimal follow-up schedules, evaluate structured transition from pediatric to adult care, assess genetic counseling and patient-navigation programmes, and determine the effectiveness and cost-effectiveness of telemedicine and registry-supported care. Greater standardization of quality-of-life assessment is also required because existing studies have used heterogeneous instruments measuring different aspects of visual function and general health (7, 15, 19, 20). Overall, the available evidence supports coordinated, multidisciplinary, lifelong, and patient-centered care, but does not establish the superiority of any single organizational model.
